# Sulfate resistance of polyvinyl alcohol and basalt fibers reinforced red mud concrete

**DOI:** 10.1371/journal.pone.0357301

**Published:** 2026-09-11

**Authors:** Fuchao Xu, Ao Liu, Zhen Liu, Zinan Fang

**Affiliations:** 1 College of Civil Engineering, Guizhou University, Guiyang, China; 2 Guizhou Electric Power Construction Supervision and Consulting Co., Ltd, Guiyang, China; Jazan University College of Engineering, SAUDI ARABIA

## Abstract

The present study investigated the reinforcement effects of polyvinyl alcohol fiber (PVAF) and basalt fiber (BF) on the performance of fiber reinforced red mud concrete (FRMC) under sulfate drying–wetting cycle. The underlying mechanisms were further explored through macroscopic performance tests in combination with pore structure, micro-morphology, and phase composition analyses. The results showed that incorporation of BF exhibited a more pronounced improvement in the sulfate resistance of FRMC compared to PVAF. Additionally, FRMC with hybrid PVAF of 0.15% and BF of 0.1% had higher sulfate resistance coefficient (0.854) after 60 cycles, showing the optimal resistance to sulfate attack. The microstructural observations showed that fibers effectively delayed the initiation and propagation of cracks under sulfate drying–wetting cycles. Thus, the fibers provided space for the accumulation of corrosion products, continuously increasing the mass of FRMC, and maintaining overall structural integrity. Pore structure analysis indicated that an appropriate amount of hybrid fibers optimized the pore structure, reducing the porosity and average pore diameter to 20.30% and 26.80 nm, respectively. Meanwhile, the synergistic effect of the fibers delayed the degradation of C-S-H gel, thereby effectively improving the sulfate resistance of FRMC.

## 1 Introduction

Currently, the field of building materials faces dual pressures of resource depletion and environmental protection. As the primary binding material in concrete, cement production is highly energy intensive and is one of the major sources of global carbon emissions [1]. Studies have shown that carbon emissions from cement production account for approximately 5%–7% of total global emissions [2], with the manufacture of one ton of ordinary Portland cement requiring about 1.7 tons of raw materials and emitting 0.5–0.6 tons of CO_2_ [3]. Under the background of “carbon neutrality” and “sustainable development,” solid waste utilization and green, low-carbon development have become critical strategic directions in China and worldwide [4–6]. Red mud (RM), a strongly alkaline solid waste generated during alumina production, is produced at a rate of approximately 1.0–2.0 tons for every ton of alumina, with an annual global output exceeding 150 million tons [7–9]. China, with its large scale aluminum industry, has accumulated an enormous amount of RM. According to data from the nonferrous metals industry, by 2024, the total accumulated stockpile of RM in China reached 115 million tons, while the comprehensive utilization rate was only 12%. The conventional wet storage method for RM not only occupies vast land resources but also poses severe environmental threats to surrounding soil, water, and ecosystems due to its strong alkalinity and trace heavy metal content [10,11]. Therefore, promoting the resource utilization of RM is both an urgent environmental demand and an important approach to developing green building materials and realizing solid waste valorization. Studies have shown that RM is rich in Fe_2_O_3_, Al_2_O_3_, and SiO_2_, and exhibits certain pozzolanic reactivity. Utilizing it as a supplementary cementitious material or filler in concrete has been recognized as one of the most effective approaches for large scale utilization [12–14]. In recent years, extensive research has been conducted worldwide on the application of RM in concrete, primarily focusing on the influence of RM content on pore structure and mechanical properties [15–17]. Yan et al. [15] found that when 20% of cement was replaced by RM, the total porosity of concrete decreased, and the 28 days (d) compressive and flexural strengths increased by 12.5% and 16.7%, respectively. However, further increasing the RM content weakened the strength. Liu et al. [16,17] reported that replacing 10%–30% of fly ash with RM slightly reduced porosity and improved strength, whereas excessive replacement (>50%) significantly increased water absorption and porosity due to the inherently porous structure of RM, leading to a sharp decline in strength. Therefore, the content of RM is a key factor determining the porosity and mechanical properties of concrete. A moderate amount of RM can provide a filling effect, thereby densifying the structure and enhancing strength. However, while high RM content concrete offers environmental and economic benefits, it also poses potential risks to its durability. Among various corrosive environments, sulfate attack is one of the primary factors affecting the durability of concrete, being both the most prevalent and destructive. Sulfate ions, which are widely present in coastal regions, saline soils, groundwater, and certain types of industrial wastewater, can penetrate into concrete and react with cement hydration products through a series of complex physicochemical processes. These reactions produce expansive products, leading to volumetric expansion, cracking, and spalling of the material, which consequently result in significant deterioration of concrete strength and stiffness, as well as a drastic reduction in service life [18,19]. Research [20] has shown that when RM replaces 40%–60% of cement, the matrix becomes more porous, facilitating the ingress, migration, and accumulation of sulfate ions, thereby accelerating the formation of expansive products such as gypsum and ettringite. This eventually results in volume expansion, cracking, and degradation of mechanical performance. Consequently, the poor sulfate resistance of high RM content concrete significantly limits its widespread engineering application.

Currently, most studies on RM concrete have focused on improving its mechanical properties [21–24], while systematic investigations of its long-term durability, particularly its resistance to sulfate attack, remain limited. Incorporating fibers into cementitious materials has been demonstrated to be an effective method to enhance sulfate resistance. Randomly distributed fibers can bridge microcracks, restraining and delaying their initiation and propagation, thereby improving the toughness, crack resistance, and overall durability of concrete [25–27]. Among various fibers, polyvinyl alcohol fiber (PVAF) and basalt fiber (BF) have attracted extensive attention due to their excellent performance in enhancing durability. PVAF exhibits outstanding toughness and ductility, as well as good hydrophilicity and strong interfacial bonding with the cement matrix, effectively controlling crack propagation, slowing degradation, and improving impermeability and durability [28,29]. BF, produced by melting and drawing natural basalt at high temperature, is a green inorganic fiber characterized by high strength, high modulus, and excellent resistance to heat, acid, and alkali [30–32], It can improve the stiffness and load bearing capacity of concrete and exhibits excellent durability under sulfate and other corrosive environments [33,34]. Wang et al. [35] reported that rubber concrete containing 0.5% PVAF exhibited lower permeability and expansion damage, thereby improving durability. Fu et al. [36] found that BF formed a stable spatial network structure through a bridging effect, and when the content was 6 kg/m^3^, the cubic compressive strength reached 47.8 MPa, with the strongest sulfate resistance. Liu et al. [37] developed a mechanical model verifying that BF delayed sulfate induced deterioration and enhanced durability. Moreover, compared with single fiber reinforcement, hybrid fiber systems typically demonstrate superior synergistic effect [38]. Recently, based on the concepts of “multi-scale reinforcement” and “hybrid synergy,” the hybridization of different fibers has emerged as an effective strategy for improving concrete performance [39]. Ran et al. [40] incorporated BF and polypropylene fiber (PPF) into rubber concrete and found that specimen containing 0.2% BF and 0.1% PPF retained high residual compressive strength after sulfate attack. Huang et al. [41] demonstrated that a combination of 0.3% BF and 0.2% PVAF optimized pore structure, limited crack development, and provided better sulfate resistance than single fibers. This “rigid–flexible hybrid” approach enables mutual enhancement at both micro and macro levels, offering an effective defense against sulfate ion attack.

Although fiber reinforcement technology has been well established in ordinary concrete, the applicability in red mud concrete still provided more discussion, particularly evaluating the influence of hybrid fiber on long-term sulfate resistance. Consequently, there is a lack of theoretical and experimental evidence supporting the use of this environmentally friendly material in sulfate rich environments. Based on previous study on the mechanical properties of high RM content (40%) concrete reinforced with PVAF and BF [42], this study further investigated the synergistic reinforcement mechanisms of fibers on the sulfate attack resistance of red mud. The complex sulfate environments were simulated through drying–wetting cycle experiments. The mass change rate, compressive strength, and sulfate resistance coefficient were compared to determine the enhancement effects of single and hybrid PVAF and BF on the sulfate resistance of RMC with 40% RM content. Furthermore, mercury intrusion porosimetry (MIP), scanning electron microscopy (SEM), and X-ray diffraction (XRD) were employed to reveal the inhibitory effects of fibers on the sulfate corrosion process, thereby elucidating the synergistic enhancement mechanism of PVAF and BF.

## 2 Materials and methods

### 2.1 Raw materials

**Fig 1:**
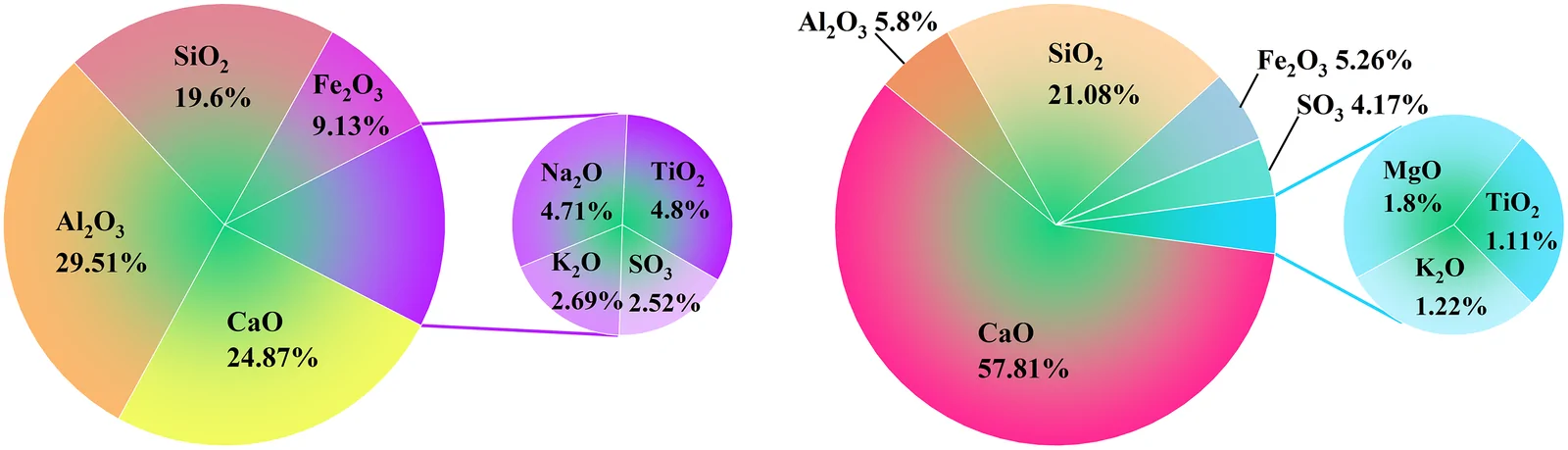
Chemical compositions of raw materials. (a) XRF of RM. (b) XRF of cement.

**Fig 2:**
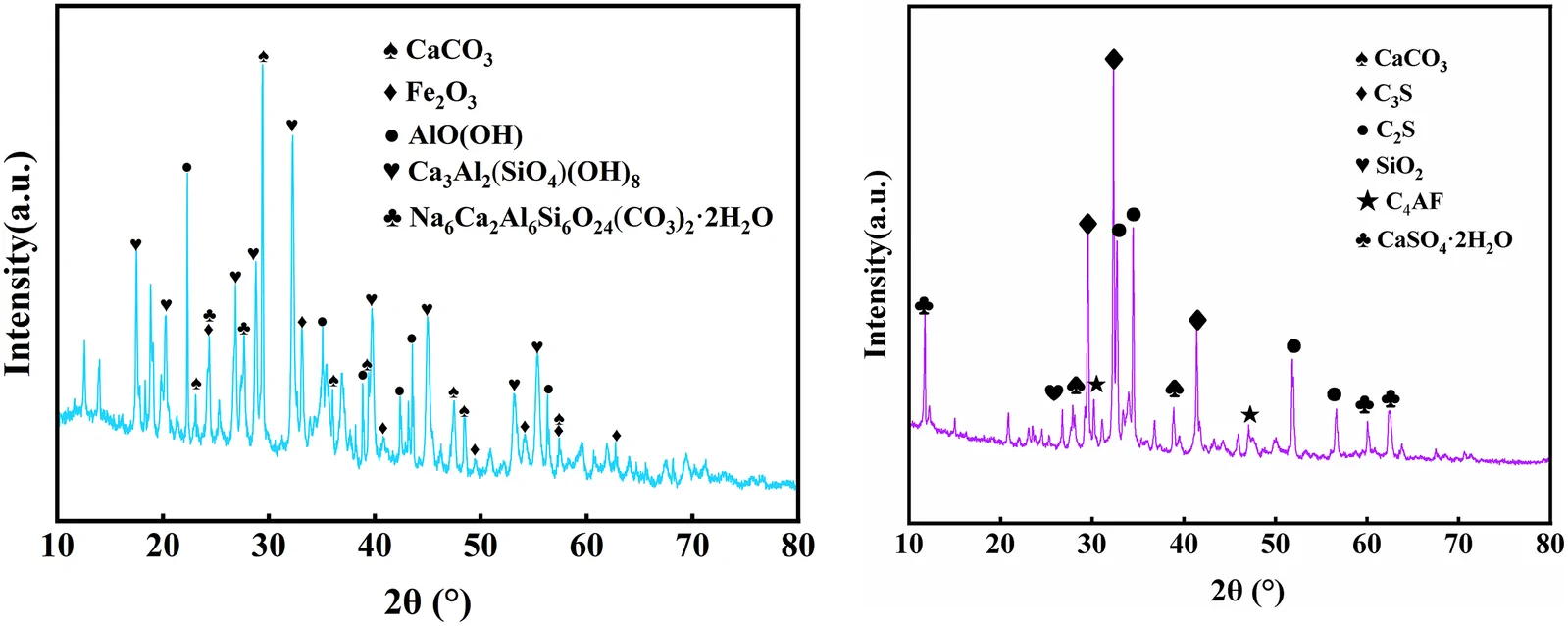
Mineral compositions of raw materials. (a) XRD of RM. (b) XRD of cement.

**Fig 3:**
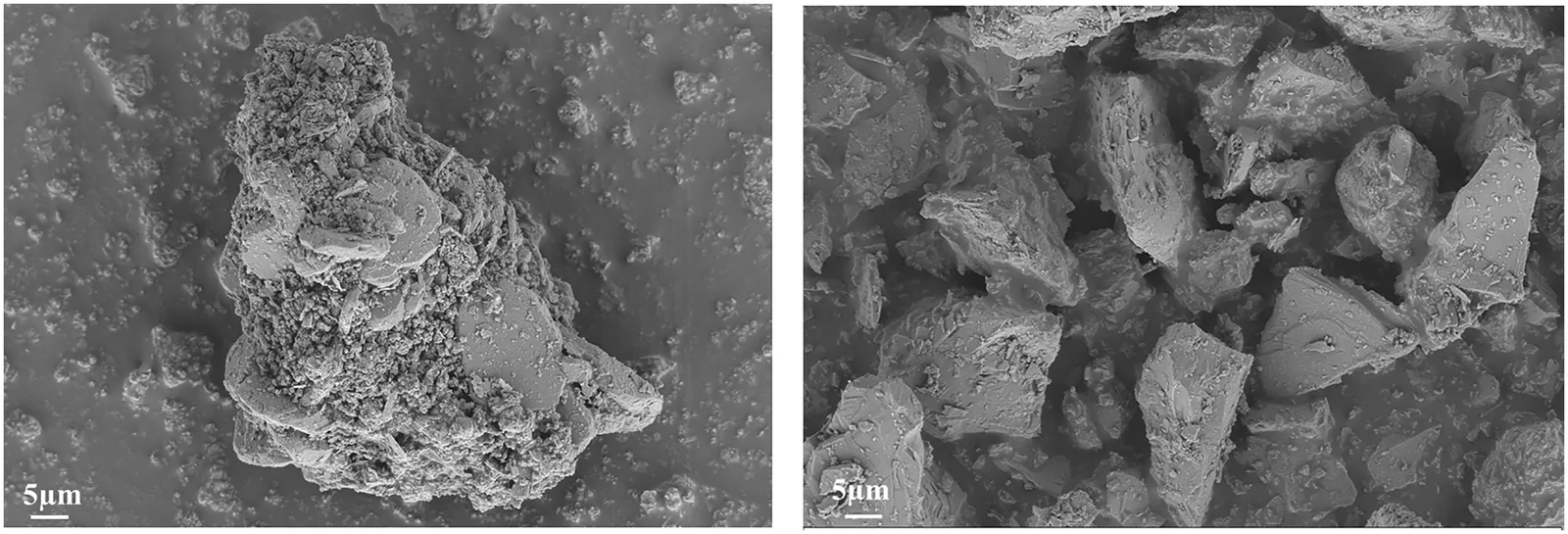
SEM image of RM and cement. (a) SEM image of RM. (b) SEM image of cement.

**Fig 4:**
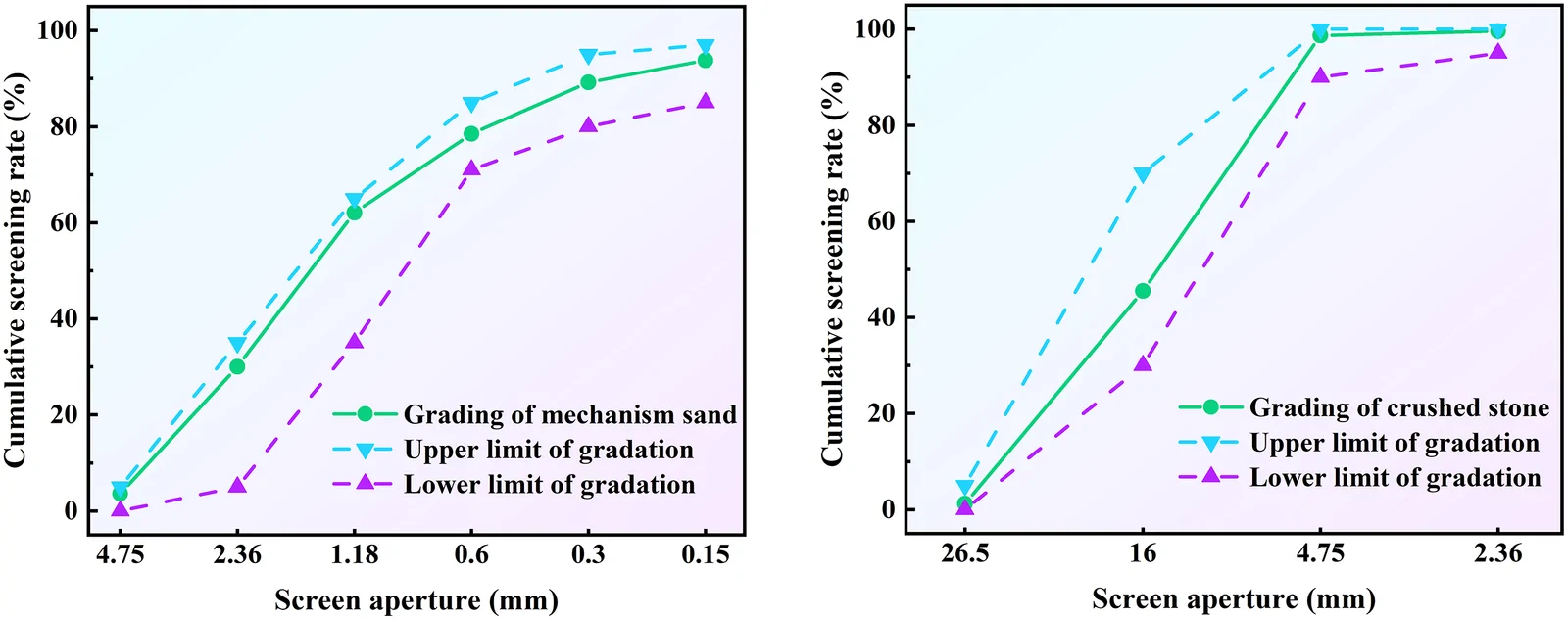
Grading curve of mechanism sand and crushed stone. (a) Grading curve of mechanism sand. (b) Grading curve of crushed stone.

The cementitious materials used in the red mud concrete (RMC) consisted of RM and cement. The RM was obtained from Guizhou Huajin Aluminum Co., Ltd, while the cement was Conch brand P·O42.5 ordinary Portland cement produced by a cement enterprise in Guizhou Province, China. The RM was subjected to simple pretreatment before being used for concrete preparation: naturally air dried RM blocks were ground in a ball mill for 5 minutes, sieved through a 0.6 mm mesh to obtain powdered RM, and then dried in an oven at 105 °C for 4 hours (h) to control its moisture content, resulting in a homogeneous RM powder. RM and cement exhibit similar chemical compositions, as evidenced by the X-ray fluorescence (XRF) results shown in Fig 1. The X-ray diffraction (XRD) patterns of RM and cement are presented in Fig 2. The main mineral phases in RM are calcite (CaCO_3_), hematite (Fe_2_O_3_), and boehmite (AlO(OH)), whereas cement is mainly composed of tricalcium silicate (C_3_S), dicalcium silicate (C_2_S), and tetracalcium aluminoferrite (C_4_AF). The microstructures of RM and cement are shown in Fig 3. RM exhibits a rough and porous surface, with numerous fine particles attached, as well as distinctive sheet-like and blocky complex structures. In contrast, the cement particles show a relatively dense surface and are mostly characterized by regular block-like morphologies. Manufactured sand was used as the fine aggregate, and crushed stone was used as the coarse aggregate, both supplied by a local materials provider in Guizhou Province. The particle size distribution curves of the two materials are shown in Fig 4. The BASF F10 melamine-based water reducing agent was produced by Shanghai Chenqi Chemical Technology Co., Ltd., with a water reduction rate of 26.2%. Two types of short cut fibers were used as reinforcing materials: polyvinyl alcohol fiber (PVAF) and basalt fiber (BF), both commercially available. The performance parameters of the two fibers are listed in Table 1.

**Table 1:** Physical and mechanical properties of fibers.

Fiber type	Length (mm)	Diameter (μm)	Density (g·cm ^− 3^)	Tensile strength (MPa)	Elastic Modulus (GPa)	Elongation (%)
PVAF	12	15.3	1.29	1830	40	7
BF	12	17	2.65	1256	76.1	3.01

### 2.2 Proportion design of concrete

In previous research [42], RMC was prepared by replacing 40wt% of cement with RM, and FRMC was produced by incorporating PVAF and BF. The volume fractions of both fibers were 0.1%, 0.15%, and 0.2%, respectively, in single fiber and hybrid combinations, resulting in a total of sixteen concrete mixtures. The mix proportions were optimized based on the mechanical performance of FRMC. On this basis, six representative groups were selected for the present durability tests to ensure systematic comparison within the experimental framework. The detailed mix proportions are provided in Table 2. Among them, RMC served as the control group, prepared with 40wt% cement replacement by RM; P0.2 and B0.2 were single fiber FRMCs prepared by adding 0.2% PVAF and 0.2% BF, respectively, to RMC; P0.1B0.1 contained 0.1% PVAF and 0.1% BF, maintaining a total fiber content of 0.2% to allow comparison with single fiber mixes; P0.15B0.1 incorporated 0.15% PVAF and 0.1% BF and achieved the highest 28 d compressive strength among all specimens; P0.2B0.2 contained 0.2% PVAF and 0.2% BF, representing the group with the highest total fiber content, used for comparative analysis within hybrid systems.

**Table 2:** Concrete mix proportion design.

Group number	Coarse aggregate (kg/m^3^)	Fine aggregate (kg/m^3^)	Cement (kg/m^3^)	RM (kg/m^3^)	Water (kg/m^3^)	Water reducing agent(kg/m^3^)	PVAF (vol%)	BF (vol%)
RMC	1017	832	228	152	171	7.6	0	0
P0.2	1017	832	228	152	171	7.6	0.2	0
B0.2	1017	832	228	152	171	7.6	0	0.2
P0.1B0.1	1017	832	228	152	171	11.4	0.1	0.1
P0.15B0.1	1017	832	228	152	171	11.4	0.15	0.1
P0.2B0.2	1017	832	228	152	171	11.4	0.2	0.2

**Fig 5:**
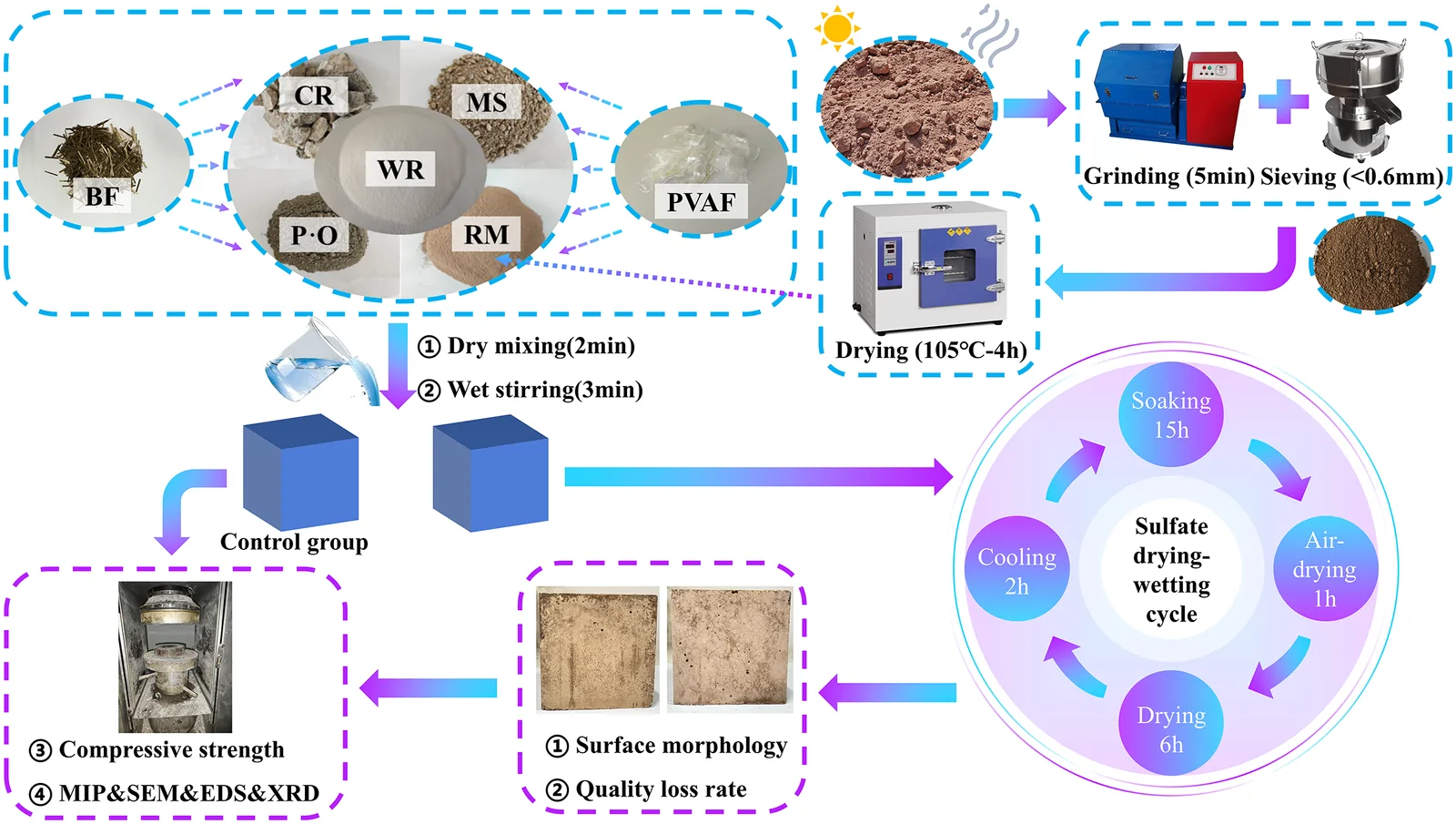
Sample preparation and test contents. The preparation process of materials required for the experiment and the manufacturing process of test specimens.

After weighing the raw materials according to the proportions in Table 2, the materials were added into a mixer, and the fibers were manually sprinkled to ensure uniform dispersion within the mixture. The dry materials were mixed for 2 minutes, followed by the addition of water and continued mixing for 3 minutes. The prepared concrete mixture was then poured into molds and compacted on a vibration table. After 24 h of molding, the specimens were demolded and placed in a stable environment with minimal temperature and humidity variation for natural curing until the designated testing ages. Cubic specimens with dimensions of 100 mm × 100 mm × 100 mm were prepared for sulfate drying–wetting cycling and control tests. The specimen preparation and testing procedure are illustrated in Fig 5.

### 2.3 Test contents and methods

#### 2.3.1 Macro testing.

The sulfate drying–wetting cycling test was conducted in accordance with the Chinese standard GB/T 50082−2009 [43]. The experiment was carried out in the Civil Engineering Experimental Center of Guizhou University. The evaluation indices included concrete surface morphology, mass change rate, compressive strength, and sulfate resistance coefficient. Before the test, specimens cured naturally for 26 d were dried in an oven at (80 ± 5) °C for 2 d to reach a constant weight. The specimens were then placed in a sulfate wet–dry cycling chamber for the sulfate attack test. A 5 wt.% Na_2_SO_4_ solution was used as the erosion medium. Each wet–dry cycle lasted 24 h: the specimens were first immersed in the solution, with the liquid level at least 20 mm above the surface of the uppermost specimens, and maintained at a constant temperature of 25–30 °C for 15 h. The solution was then drained from the chamber, and the specimens were air-dried naturally for 1 h, followed by drying at (80 ± 5) °C for 6 h. After drying, the fan inside the chamber was activated to provide forced-air cooling for 2 h. Completion of the above procedures constituted one full wet–dry cycle, and the test was conducted continuously according to this cycling regime. The specific procedure is illustrated in Fig 5. One complete cycle consisted of the entire process from immersion to cooling. A total of 15, 30, 45, and 60 cycles were conducted, with the sulfate solution renewed every month to maintain a stable concentration. After every 15 cycles, the surface morphology, mass, and compressive strength of the specimens were recorded.

The compressive strength of the concrete was measured using a CXYAW-2000S compression testing machine and RMT device at a loading rate of 0.5 MPa/s. The compressive strength specimens were non-standard specimens; therefore, the experimental results were multiplied by a size conversion coefficient of 0.95. Three specimens were tested in each group, and the average value was taken as the compressive strength. The formula for calculating compressive strength of concrete is as follows:


$$\begin{array}{c} f_{\text{c0}}=\text{0}.\text{95}\cdot (F_{\text{c0}}/A) \end{array}$$ (1)

$$\begin{array}{c} f_{\text{cn}}=\text{0}.\text{95}\cdot (F_{\text{cn}}/A) \end{array}$$ (2)

Where: *f*_c0_ and *f*_cn_: Cubic compressive strength (MPa); *F*_c0_ and *F*_cn_: Failure load (N); *A*: Bearing area (mm^2^).

The corrosion resistance coefficient of concrete was used to characterize the ability of the material to retain its mechanical properties under corrosive environments. A higher value indicates better corrosion resistance. It is calculated as follows:


$$\begin{array}{c} K_{\text{c}}=f_{\text{cn}}/f_{\text{c0}} \end{array}$$ (3)

Where: *K*_c_: Sulfate resistance coefficient; *f*_cn_: Compressive strength of specimens after n drying–wetting cycles (MPa); *f*_c0_: Compressive strength of specimens under natural curing at the same age (MPa).

The mass change rate of concrete was used to characterize the degree of mass variation of the specimens during sulfate attack, and its value reflects the deterioration degree of the material. It is calculated as follows:


$$\begin{array}{c} M_{\text{f}}=((m_{\text{c}\text{n}}-m_{\text{c}\text{0}})/m_{\text{c0}})\times \text{100}\% \end{array}$$ (4)

Where: *M*_f_: Mass change fraction (%); *m*_cn_: Mass of concrete specimens after n cycles (kg) (The data were obtained from the same specimen tested at different cycling stages); *m*_c0_: initial mass of the same specimen before sulfate attack (kg).

#### 2.3.2 Microstructure and chemical characterization.

To investigate the influence of PVAF and BF on FRMC under sulfate attack, fractured concrete fragments after the test were prepared for microstructural analyses. Capillary pore characterization was conducted using a Micromeritics AutoPore V 9620 mercury intrusion porosimeter (MIP) from the United States, with a pressure range of 0.2–30,000 psi and an analyzable pore diameter range of 5–800,000 nm. MIP samples were extracted from the mortar enriched central region of the failed compressive specimens to ensure a representative reflection of the overall pore structure of the concrete. The fracture surfaces of selected specimens were examined using a ZEISS Sigma 360 scanning electron microscope (SEM), from Germany to observe the microstructural characteristics of the fiber–matrix interfaces. The phase composition of RMC and FRMC was analyzed by X-ray diffraction (XRD). XRD samples were taken from the central portion of the failed specimens, with visible aggregates removed and the remaining mortar ground into powder to ensure accuracy in analyzing the matrix phase composition. The XRD instrument used was a Rigaku SmartLab SE from Japan, operated at a scanning speed of 5°/min and a diffraction angle range of 10°–80°.

## 3 Results and discussion

### 3.1 Flowability test

**Fig 6:**
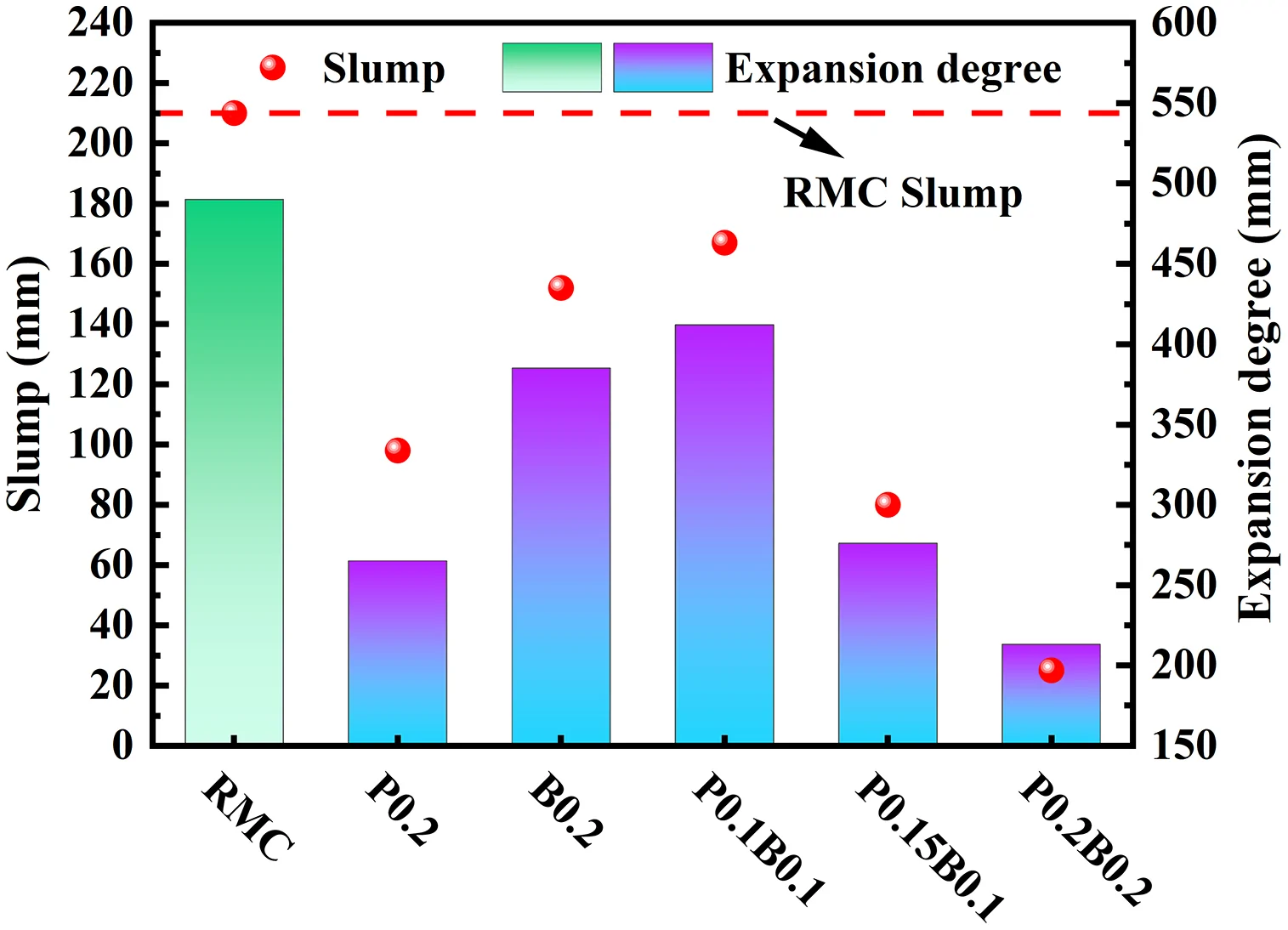
Slump and expansion degree values of RMC and FRMC.

To evaluate the effect of fiber incorporation on the workability of concrete, the flowability results of specimens with different mix proportions were further analyzed, as shown in Fig 6. The relevant data were obtained from the authors’ previous study, and the results for six mixtures, namely RMC, P0.2, B0.2, P0.1B0.1, P0.15B0.1, and P0.2B0.2, were extracted and uniformly plotted [42]. The results indicate that the reference group RMC exhibited a relatively high flowability, whereas fiber incorporation reduced the workability of the fresh mixture, with a more pronounced loss of flowability observed in the hybrid fiber system. This can mainly be attributed to the distribution characteristics and interlacing effect of fibers within the paste, which increase the resistance to particle movement and enhance the cohesiveness of the system. During mixture preparation, the dosage of the water-reducing agent was appropriately adjusted to ensure adequate moldability and maintain comparable initial workability among the specimens. Specifically, a dosage of 2% was used for the reference group and single-fiber groups, while the dosage was uniformly increased to 3% for the hybrid fiber groups. This adjustment was mainly intended to compensate for the significant flowability loss caused by the incorporation of hybrid fibers. Under this condition, the dosage of the water-reducing agent remained consistent among the hybrid fiber mixtures, thereby ensuring a reliable basis for comparison between different hybrid fiber proportions. It should be noted that variations in the dosage of the water-reducing agent may affect paste dispersion, pore structure characteristics, and mechanical properties to some extent. Therefore, when comparing different systems, this factor may be coupled with the fiber effect. Nevertheless, the mechanical properties and MIP results in this study (see Sections 3.4 and 4.1) show that, within an appropriate fiber dosage range, such as P0.15B0.1, both the pore structure and strength exhibited an improving trend, and this variation corresponded well with the fiber dosage and fiber combination. Within the hybrid fiber system, because the dosage of the water-reducing agent was kept constant, the performance differences among different mixtures remain highly comparable. Based on these results, it can be considered that, within the scope of this study, the changes in material performance were mainly governed by fiber parameters, while the influence of differences in water-reducing agent dosage was relatively limited.

### 3.2 Morphological evolution

**Fig 7:**
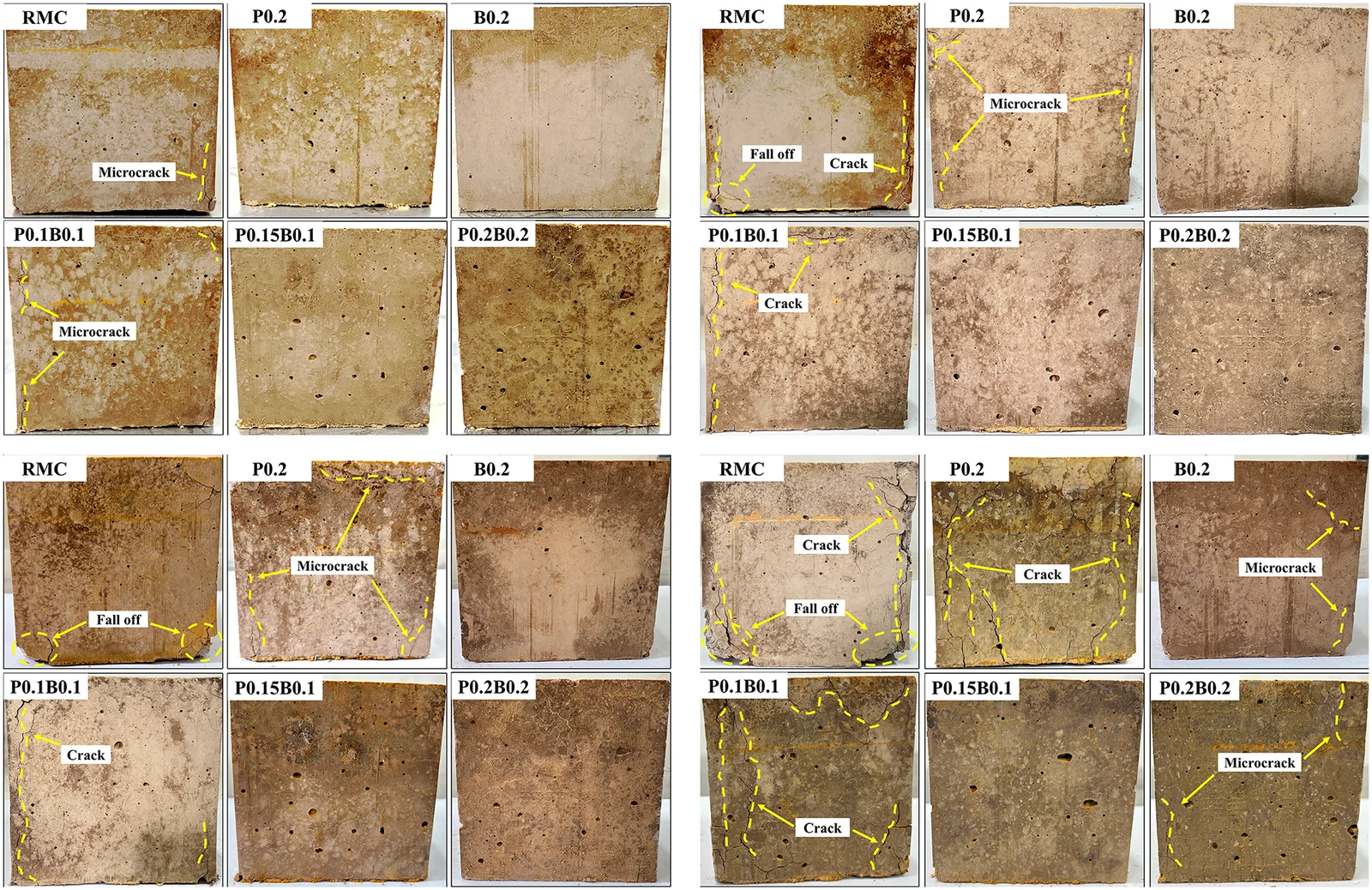
Surface morphology of RMC and FRMC after sulfate attack. (a) 15 cycles. (b) 30 cycles. (c) 45 cycles. (d) 60 cycles.

The surface morphological evolution of RMC and FRMC specimens under different numbers of sulfate drying–wetting cycles is shown in Fig 7. With the increase in cycle numbers, all specimens exhibited varying degrees of surface deterioration. After 15 cycles, microcracks appeared at the edges and corners of the RMC specimen, and although its overall shape remained intact, obvious cracks and mortar fall off were observed after 30 cycles. As the number of cycles increased, the deterioration intensified, resulting in poor structural integrity. After 60 cycles, the cracks extended across the surface with severe scaling and a highly loose structure, indicating the poorest sulfate resistance. In contrast, the P0.2 specimen mainly exhibited the propagation of microcracks before 45 cycles, and although wider cracks and slight loosening occurred after 60 cycles, no noticeable spalling was observed, and the overall structure remained intact. The incorporation of PVAF delayed crack development and structural deterioration to some extent, but its long-term durability improvement remained limited. The B0.2 specimen demonstrated relatively stable performance throughout the entire cycling process, except for minor localized cracking after 60 cycles, its surface largely remained intact without evident erosion or penetrating cracks, indicating that BF exerted a significant reinforcing effect on the sulfate resistance of FRMC. Therefore, the fiber type played a critical role in crack control and interfacial protection. Among the hybrid fiber specimens, P0.1B0.1 behaved similarly to RMC, with microcracks emerging as early as 15 cycles. After 60 cycles, the number of cracks and the extent of deterioration were both significantly higher than those in the single fiber specimens, and the overall structure became relatively loose. In contrast, the P0.15B0.1 specimen maintained excellent surface integrity throughout the cycling process, and even after 60 cycles, no apparent microcracks were observed, exhibiting the best performance among all groups. The P0.2B0.2 specimen developed a few microcracks after 60 cycles. However, its degree of deterioration was considerably lower than that of RMC and the single fiber specimens, and also better than P0.1B0.1. This behavior could be attributed to the synergistic effect of appropriately increased fiber content, which effectively inhibited crack propagation [44]. In summary, RMC exhibited poor apparent durability under sulfate drying–wetting cycling and was inadequate for service in high sulfate environments. The incorporation of PVAF and BF substantially improved the apparent durability of FRMC. However, the enhancement effect was governed by the coupled influence of fiber type and content. The single incorporation of BF was more effective than PVAF, while a proper hybrid ratio (P0.15B0.1) yielded a more pronounced reinforcing effect in terms of microcrack control, interfacial protection, and structural integrity maintenance.

### 3.3 Mass change fraction

**Fig 8:**
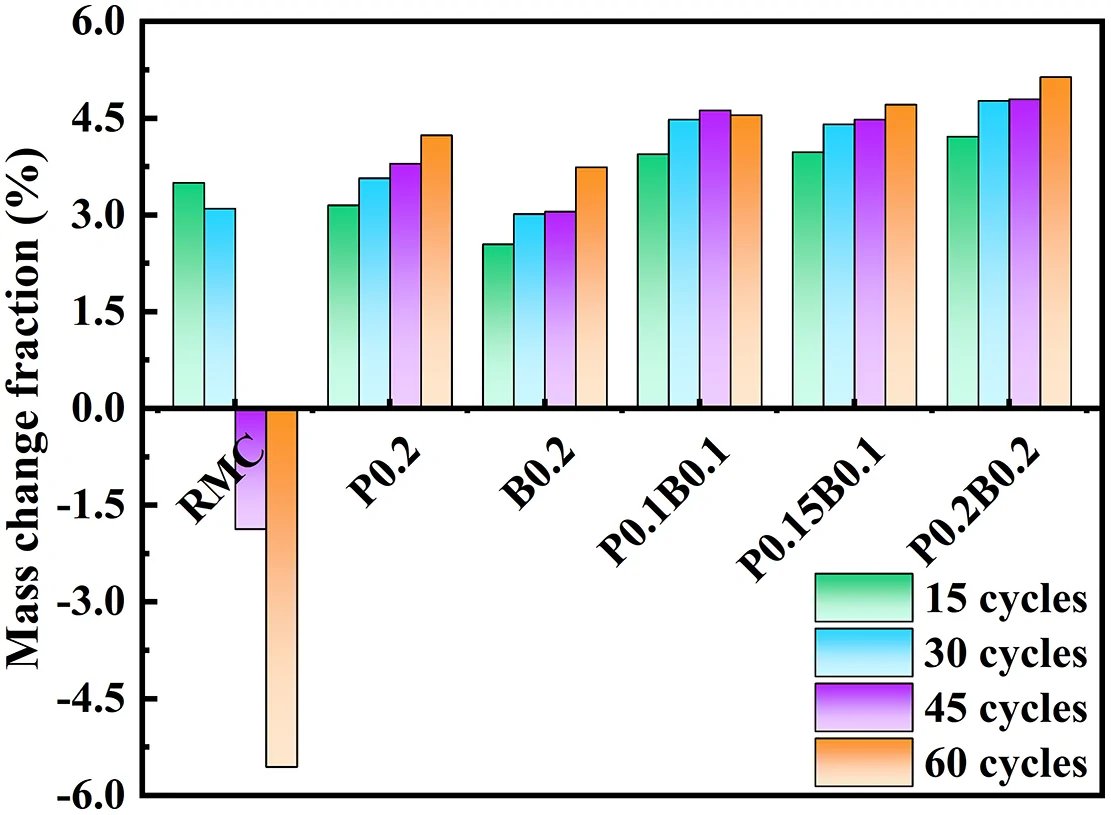
Mass change fraction of RMC and FRMC after sulfate attack.

The mass change fractions of RMC and FRMC specimens under different sulfate drying–wetting cycles are presented in Fig 8. Overall, the mass of all specimens increased during the initial cycling stage. This was attributed to the ingress of sulfate ions into the concrete interior, where they reacted with hydration products of cement to form expansive compounds such as gypsum and ettringite (AFt). These products partially filled the capillary pores, enhanced the matrix compactness, and consequently led to mass gain [45]. As the exposure continued, some specimens exhibited a reduced growth rate or even a decline in mass due to the progressive internal damage, where loose corrosion products or aggregates were eroded, resulting in aggravated deterioration [46]. Specifically, the mass of RMC increased slightly during the early cycles but declined as the number of cycles increased because of structural degradation. After 45 cycles, the mass decreased by 1.87% compared with the initial value, and after 60 cycles, severe structural damage and spalling of concrete fragments led to substantial mass loss. This phenomenon was related to the high alkalinity of RM in the matrix, which promoted SO_4_^2-^ diffusion and accelerated the formation of AFt-type corrosion products, resulting in volume expansion and matrix disruption [47]. In contrast, both P0.2 and B0.2 specimens exhibited more favorable mass gain trends, reaching their maximum mass change at 60 cycles, with increases of 4.24% and 3.74% relative to their initial mass, respectively. It is noteworthy that the mass gain of P0.2 consistently exceeded that of B0.2. This was because the PVAF surface was rich in polar groups [48], which could adsorb SO_4_^2-^ and other corrosive ions during exposure while also absorbing and retaining more sulfate solution through capillary pores. These processes facilitated further hydration reactions, generating additional gypsum and AFt. The deposition of these products within pores and microcracks substantially increased the specimen mass. Conversely, BF possessed higher chemical stability and lower hydrophilicity, exhibiting weaker ion adsorption and denser intrinsic structure, which effectively hindered the further diffusion of sulfate ions. Therefore, fewer corrosion products formed, resulting in a smaller mass increase. For the hybrid fiber specimens, P0.1B0.1 exhibited relatively stable mass change throughout the entire process. However, after 30 cycles, the rate of mass increase slowed, and by 60 cycles, minor mortar spalling caused a slight decrease in mass, although it remained 4.55% higher than the initial value. This indicated that at low fiber contents, the hybrid system could not completely suppress the accumulation of structural damage under long-term sulfate exposure, leading to limited durability improvement. In contrast, P0.15B0.1 and P0.2B0.2 exhibited better mass gain behavior, both maintaining an increasing trend even after 60 cycles, with mass increases of 4.72% and 5.14%, respectively. The higher mass change observed in P0.2B0.2 could be attributed to the inevitable increase in porosity associated with higher fiber content [49], which provided more permeation channels for sulfate ions, leading to the formation of more corrosion products and thus greater mass gain. Nevertheless, the mass growth of P0.15B0.1 was more stable and without significant fluctuations, owing to the optimized pore size distribution induced by an appropriate fiber content, which yielded a denser structure and effectively limited the diffusion depth of sulfate ions [50]. Overall, the hybrid fiber specimens exhibited greater mass gain than both the single fiber and RMC specimens. This was because the hybrid fiber effectively controlled microcrack propagation, allowing the internal structure of the specimens to remain intact for a longer period, thereby providing more space and favorable conditions for the formation and deposition of expansive products such as gypsum and AFt, resulting in continuous mass increase of the specimens.

### 3.4 Compressive strength

**Fig 9:**
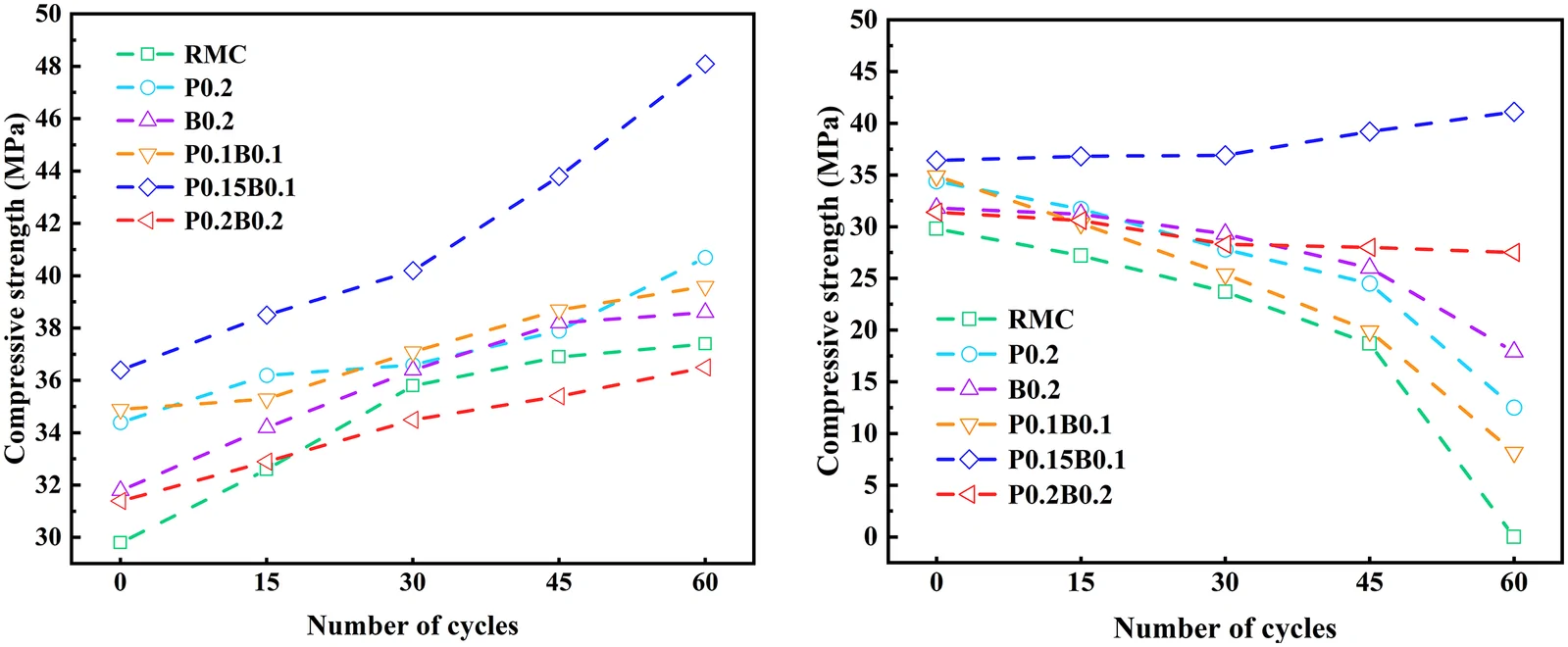
Compressive strength under different numbers of wetting-drying cycles. (a) Natural curing. (b) Sulfate attack.

The variations in compressive strength of RMC and FRMC under natural curing and sulfate attack are illustrated in Fig 9. As shown in Fig 9(a), under natural curing conditions, the compressive strength of both RMC and FRMC increased continuously with curing age, indicating that hydration reactions proceeded steadily, hydration products gradually filled the pores, and the matrix compactness improved, resulting in enhanced compressive strength. At 0 cycles (These data were obtained from a previous study on 28 d compressive strength [42])), the compressive strength of RMC was the lowest, only 29.8 MPa, primarily due to the low pozzolanic activity of RM and the insufficient formation of hydration products. In contrast, the compressive strengths of FRMC with PVAF and BF were 34.4 MPa and 31.8 MPa, representing increases of 15.4% and 6.7% compared with RMC, respectively. The incorporation of fibers contributed to the strengthening of the matrix, in which the strong interfacial bonding of PVAF and the rigid support effect of BF both played positive roles [51,52]. Among the hybrid fiber groups, the compressive strengths of P0.1B0.1, P0.15B0.1, and P0.2B0.2 reached 34.9 MPa, 36.4 MPa, and 31.4 MPa, corresponding to increases of 17.1%, 22.1%, and 5.4% relative to RMC, indicating that a proper combination of fibers could further enhance the compressive strength of FRMC. Particularly, P0.15B0.1 maintained excellent compressive performance at all ages, reaching a maximum of 48.1 MPa at 60 cycles, 28.6% higher than RMC. In contrast, the compressive strength of P0.2B0.2 dropped below that of RMC after 30 cycles, and decreased to only 36.5 MPa after 60 cycles, representing a 2.4% reduction. This was mainly attributed to the adverse effects induced by excessive fiber content: fiber agglomeration increased porosity, fiber hydrophilicity weakened local hydration reactions, and numerous fiber–matrix interfaces reduced overall integrity. Consequently, as microcracks propagated and internal defects accumulated, both compactness and load-bearing capacity significantly declined in the later stages.

As shown in Fig 9(b), the compressive strength of RMC and FRMC under sulfate drying–wetting cycles exhibited distinct degradation patterns. Except for P0.15B0.1, all groups showed varying degrees of strength deterioration, reflecting the continuous destructive effects of sulfate attack on the matrix structure. RMC exhibited the poorest corrosion resistance, after 60 cycles, the specimen was severely damaged and could not be tested for compressive strength, which was thus regarded as 0 MPa. In the single fiber groups, the strengths of P0.2 and B0.2 declined slowly within the first 45 cycles, indicating that the crack bridging function of fibers delayed structural degradation to some extent. However, after 60 cycles, their compressive strengths sharply dropped to 12.5 MPa and 17.9 MPa, representing reductions of 63.7% and 43.7% from the initial values, suggesting that single fiber provided limited long-term corrosion resistance. The P0.1B0.1 group exhibited a similar early-stage trend to the single fiber groups, but its strength declined more rapidly after 30 cycles, even falling below that of P0.2 and B0.2. After 60 cycles, its compressive strength was only 8.2 MPa, a 76.5% reduction, implying that low fiber content failed to produce an effective synergistic toughening effect and was insufficient to resist severe deterioration. In contrast, the P0.15B0.1 group exhibited a continuous increase in strength throughout the cycles, reaching 41.1 MPa after 60 cycles,12.9% higher than the initial value, demonstrating outstanding sulfate resistance. This phenomenon does not indicate continuous strengthening of the material under sulfate attack, but more likely reflects a staged densification effect during the erosion process [53]. On the one hand, under wet–dry cycling conditions, the incompletely hydrated cementitious materials inside the specimens continue to undergo hydration reactions, promoting gel formation and thereby improving the compactness of the matrix. On the other hand, products such as gypsum and AFt formed during the early stage of sulfate attack can fill pores and microcracks, which improves the structural compactness of the material to some extent and enhances its load-bearing capacity. In addition, the effective crack-constraining effect of the hybrid fiber system allows the erosion products to mainly act as fillers rather than destructive agents, thereby delaying strength degradation. Meanwhile, owing to the combined effects of the increased total content of hybrid fibers and the synergistic action between PVAF and BF, crack suppression and stress dispersion were enhanced, thereby effectively delaying the structural failure process. It is noteworthy that although P0.2B0.2 exhibited lower compressive strength than RMC under natural curing, it maintained relatively high strength stability under sulfate attack, reaching 27.5 MPa after 60 cycles, with only a 12.4% reduction from the initial value. This indicates that high fiber content hybrid system demonstrates a certain adaptability in sulfate environments.

### 3.5 Sulfate resistance coefficient

**Fig 10:**
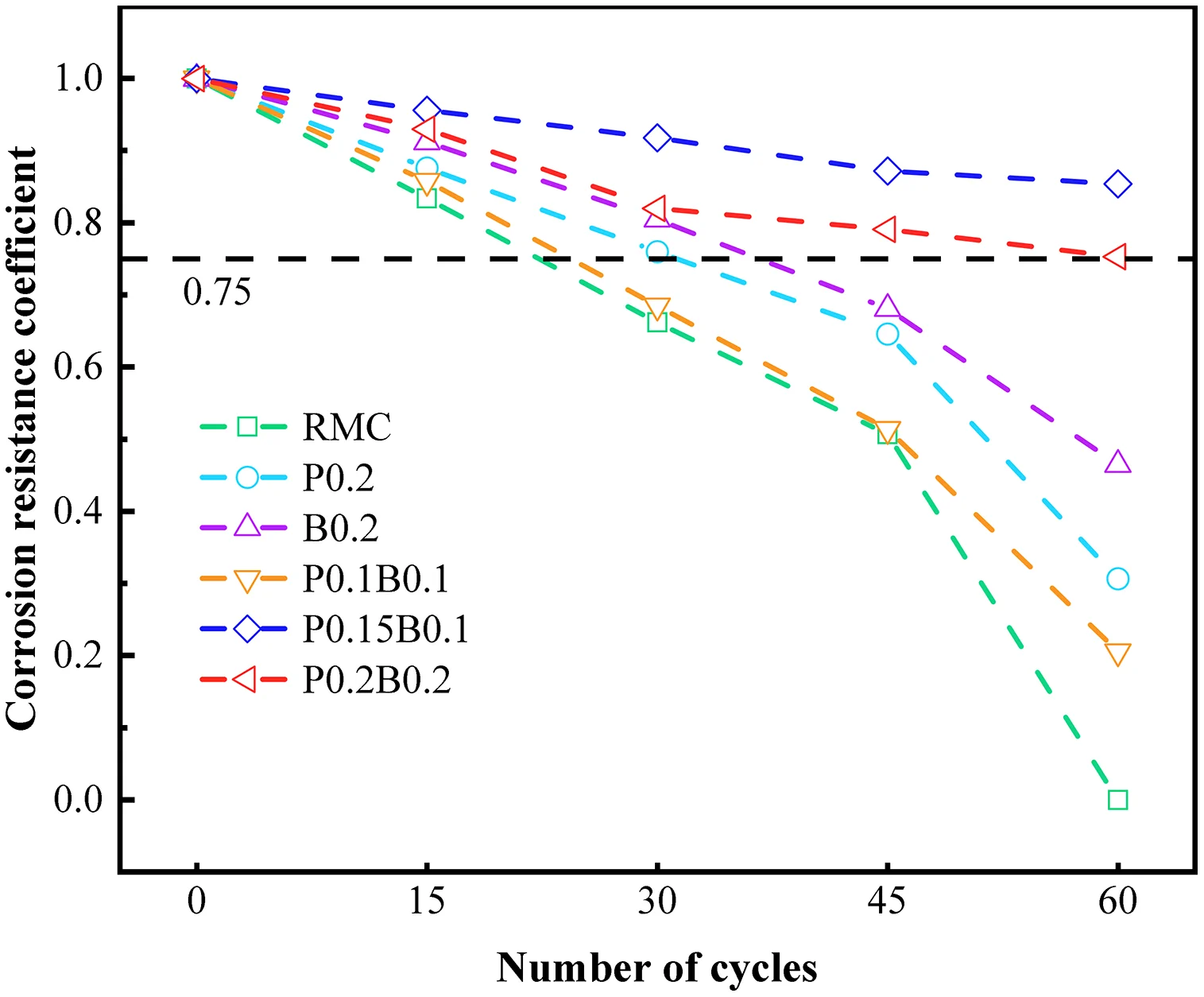
Sulfate resistance coefficients of RMC and FRMC.

The evolution of the sulfate resistance coefficient of RMC and FRMC during sulfate drying–wetting cycles is shown in Fig 10. Overall, the sulfate resistance coefficients of all groups decreased with increasing cycle number, but the rate of reduction varied considerably. RMC exhibited the weakest corrosion resistance, after 15 cycles, its coefficient had dropped to 0.834, and further decreased to 0.662 after 30 cycles, falling below the KS30 sulfate resistance threshold of 0.75 [43]. After 60 cycles, the specimen was severely damaged and almost completely lost its load bearing capacity. This deterioration was attributed to the strong alkalinity of RM, which accelerated the formation of expansive corrosion products and caused severe internal degradation. In the single fiber groups, the sulfate resistance coefficients of P0.2 and B0.2 after 15, 30, 45, and 60 cycles were 0.876, 0.76, 0.646, and 0.307, and 0.912, 0.805, 0.681, and 0.465, respectively. Both groups followed a similar trend, showing a relatively slow decrease in the early stage. The introduction of fibers effectively delayed the penetration of corrosive agents and improved the early resistance of FRMC to deterioration. However, after 45 cycles, their coefficients had dropped below 0.75, and the rate of decline accelerated thereafter, suggesting that although fibers improved durability to some extent, their long-term enhancement effect remained limited. The evolution of sulfate resistance in hybrid fiber groups was more complex and strongly affected by fiber content. The P0.1B0.1 group exhibited a sulfate resistance coefficient of 0.858 after 15 cycles, comparable to that of single fiber groups, but it rapidly dropped below 0.75 after 30 cycles, followed by pronounced deterioration in the later stage. In contrast, the P0.15B0.1 group consistently maintained a high sulfate resistance coefficient throughout the test, reaching 0.854 after 60 cycles, showing excellent resistance to sulfate attack. The P0.2B0.2 group ranked second, with a coefficient of 0.753 after 60 cycles, still at a relatively high level. The differences in sulfate resistance performance were mainly attributed to the effect of fiber content on structural stability. At low fiber contents, the reinforcement network was insufficient to maintain integrity, leading to interfacial degradation and accelerated ion ingress in the later stages. With moderate fiber addition, the synergistic interaction between fibers was maximized, effectively stabilizing the pore structure and improving erosion resistance. Although excessive fiber content introduced some void defects, the overall structure still provided effective protection against sulfate attack.

## 4 Fiber reinforcement mechanism

### 4.1 Micropore structure analysis based on MIP

The pore structure characteristics of RMC and FRMC after 60 cycles of sulfate drying–wetting were analyzed using MIP, along with naturally cured RMC of the same age (RMC-0). Table 3 lists the porosity and average pore diameter of each group. The unexposed RMC-0 exhibited a porosity of 19.56% and an average pore diameter of 38.55 nm, indicating a relatively compact pore structure. After 60 cycles of sulfate drying–wetting, the porosity of RMC increased to 23.10%, while the average pore diameter decreased to 34.44 nm. This was attributed to the increase in the number of pores within the matrix during erosion, accompanied by the partial filling of microcracks and capillary channels by gypsum and AFt, leading to higher porosity but smaller average pore size [54]. In the fiber reinforced groups, the incorporation of either PVAF or BF increased both the porosity and the average pore diameter. This can be explained by the introduction of a highly porous interfacial transition zone (ITZ) [49,55], which promoted ion penetration during cyclic exposure. Notably, the P0.2 group exhibited the highest porosity but a smaller average pore size than the B0.2 group. This behavior was mainly due to the superior crack bridging capacity of PVAF compared with BF. However, its strong adsorption ability facilitated the accumulation of expansive products on its surface, which aggravated pore deterioration and resulted in poorer corrosion resistance. Among the hybrid fiber systems, the P0.1B0.1 mixture exhibited the highest porosity (26.17%) and the largest average pore diameter (59.87 nm), both significantly greater than those of the other groups. As the hybrid fiber content increased, the pore structure was noticeably optimized. Specifically, P0.15B0.1 presented the lowest porosity (20.30%) and the smallest average pore diameter (26.80 nm), indicating the most refined pore structure and the most effective resistance to sulfate induced damage.

**Table 3:** Porosity and average pore diameter.

Parameters	Sample Groups						
	RMC-0	RMC	P0.2	B0.2	P0.1B0.1	P0.15B0.1	P0.2B0.2
Porosity (%)	19.56	23.10	27.52	23.59	26.17	20.30	21.85
Average pore diameter (nm)	38.55	34.44	39.05	41.59	59.87	26.80	38.37

**Fig 11:**
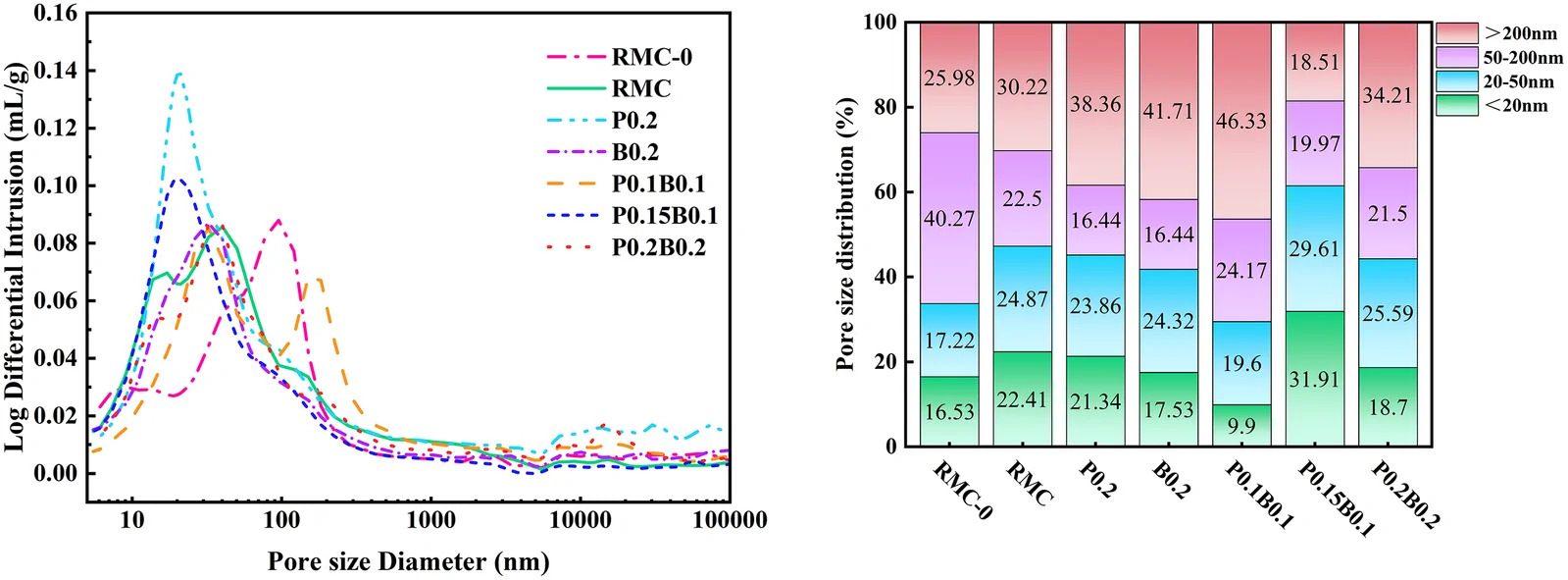
MIP test results of specimens. (a) Pore size distribution. (b) Proportion of different types of pores.

Fig 11 further illustrates the pore size distribution characteristics and the proportion of different types of pores for each specimen. The majority of pores in all groups were smaller than 200 nm. Based on their effect on matrix durability, pores can be categorized as harmless pores (<20 nm), less harmful pores (20−50 nm), harmful pores (50−200 nm), and multiple harmful pores (>200 nm) [56]. Compared with RMC-0, the sulfate attacked RMC showed significant changes in pore size distribution, with simultaneous increases in the proportions of both multiple harmful and harmless pores. The proportion of harmless pores increased from 16.53% to 22.41%, while that of multiple harmful pores increased from approximately 25.98% to 30.22%. This suggested that, during erosion, crack formation and large pore generation occurred alongside the filling of capillary pores by corrosion products, leading to a redistribution of the pore structure. Both PVAF and BF reinforced specimens exhibited higher proportions of multiple harmful pores and lower proportions of harmless and less harmful pores. The proportion of multiple harmful pores increased from 30.22% to 38.36% and 41.71%, respectively; the proportion of less harmful pores decreased from 24.87% to 23.86% and 24.32%, respectively; and the proportion of harmless pores decreased from 22.41% to 21.34% and 17.53%, respectively. Although the proportion of multiple harmful pores in B0.2 was slightly higher than that in P0.2, the P0.2 group displayed a broader and more right shifted distribution curve in the medium to large pore range, indicating higher pore connectivity and more severe degradation. In the hybrid fiber systems, the low fiber content group P0.1B0.1 had the highest proportion of multiple harmful pores (46.33%) and the lowest proportion of harmless pores (9.9%), with a pronounced peak beyond 100 nm. This was attributed to the insufficient synergistic interaction between fibers, which induced interfacial defects and led to pore structure deterioration. In contrast, the moderate hybrid fiber content P0.15B0.1 group exhibited the most favorable pore structure: a significantly higher proportion of harmless pores, a much lower proportion of multiple harmful pores, The proportion of harmless pores increased to 31.91%, while that of multiple harmful pores decreased to 18.51%, representing an increase of 9.50 percentage points and a decrease of 11.71 percentage points, respectively, compared with RMC. and an overall left shifted and narrowed pore size distribution curve. Owing to the increased total content of hybrid fibers and the synergistic effect between PVAF and BF, the pore structure was effectively optimized, and the formation of large pores was significantly inhibited. The proportions of harmless pores and multiple harmful pores were 18.70% and 34.21%, respectively, better than the single fiber groups but inferior to P0.15B0.1group. These results demonstrate that the fiber ratio had a pronounced influence on the sulfate resistance of FRMC.

### 4.2 Microstructure analysis based on SEM and EDS

**Fig 12:**
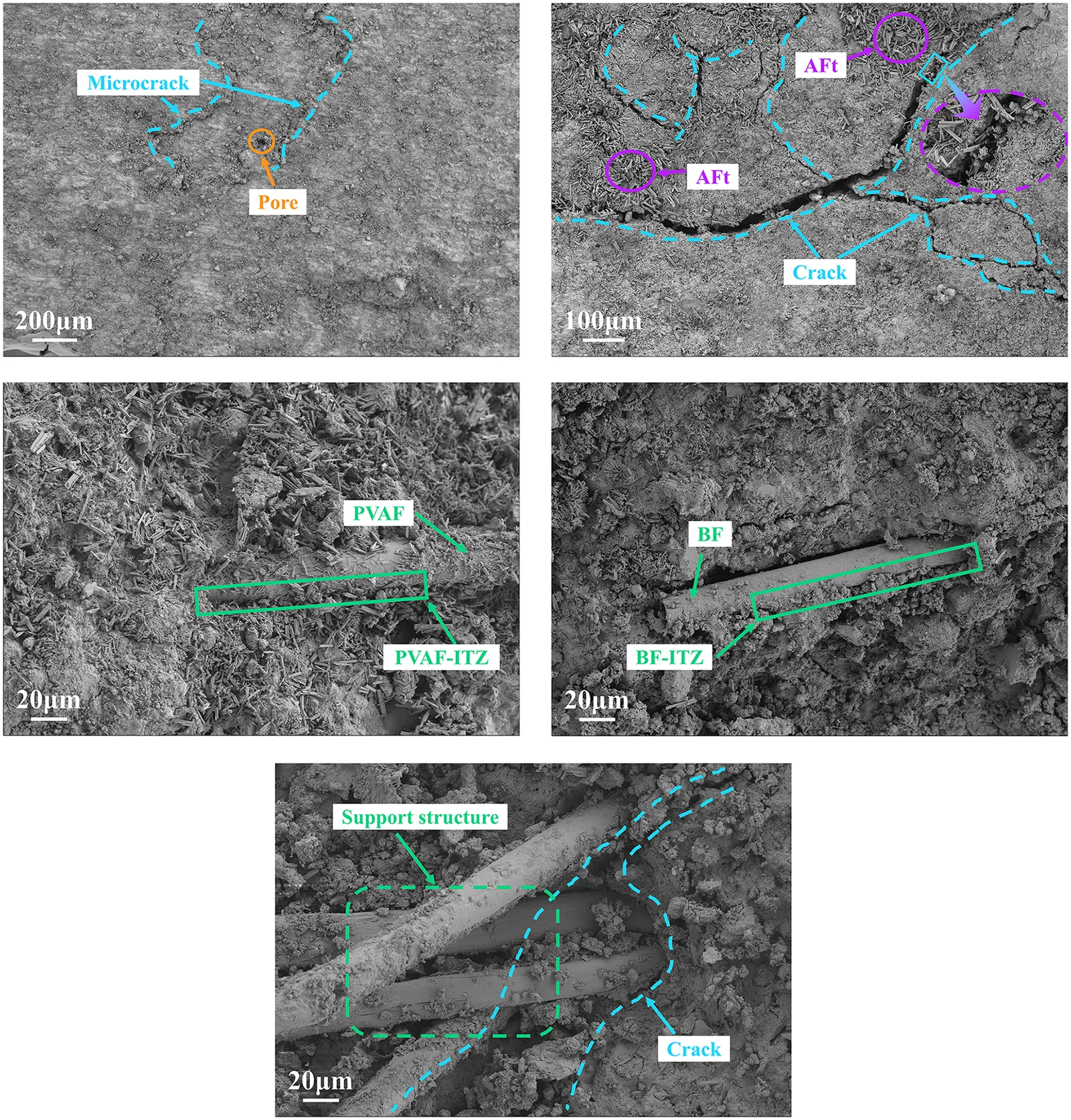
SEM images of RMC and FRMC after 60 sulfate drying-wetting cycles. (a) RMC-0. (b) RMC. (c) PVAF-ITZ. (d) BF-ITZ. (e) Support structure.

To elucidate the mechanism by which fibers influence the sulfate drying–wetting resistance of RMC and FRMC, the microstructure of specimens was examined using scanning electron microscopy (SEM), and elemental point analysis on fiber surfaces was conducted through energy dispersive spectroscopy (EDS). As shown in Fig 12(a), the internal structure of unexposed RMC (RMC-0) appeared relatively dense, though initial microcracks and pores were still visible, which accounted for its insufficient strength. Fig 12(b) presents the microstructure of RMC after 60 cycles, revealing numerous wide and deep penetrating cracks on the matrix surface. These cracks intensified the erosion reaction, leading to the abundant formation of needle-like AFt around the crack regions. In the early stage of erosion, SO_4_^2-^ ions reacted with hydration products within the matrix to form gypsum and AFt, which filled original pores and increased compactness. This explained the early-stage mass gain observed in both RMC and FRMC. However, with continued accumulation of corrosion products, expansive stresses developed within cracks, resulting in their propagation and matrix deterioration [45]. Notably, the high RM content in this study increased the Al content and alkalinity of the matrix, both of which promoted the preferential formation of AFt with a higher reaction rate [57]. Consequently, AFt rather than gypsum was identified as the dominant corrosion product. In the fiber reinforced groups, as shown in Fig 12(c) and 12(d), abundant corrosion products were attached to the surfaces of both PVAF and BF, leading to tighter bonding between fibers and the matrix and optimizing the fiber matrix interfacial transition zone (ITZ). When cracks formed, the fibers engaged in strong mechanical interlocking with the matrix, effectively restraining crack propagation and mitigating strength degradation. Furthermore, as shown in Fig 12(e), both PVAF and BF were evenly dispersed within the matrix, forming a reticulated support structure. This structure effectively suppressed the propagation of microcracks induced by expansive stress into through cracks, reduced the risk of spalling, and maintained specimen integrity. Therefore, an appropriate hybridization of fibers effectively improved the sulfate resistance of FRMC.

**Fig 13:**
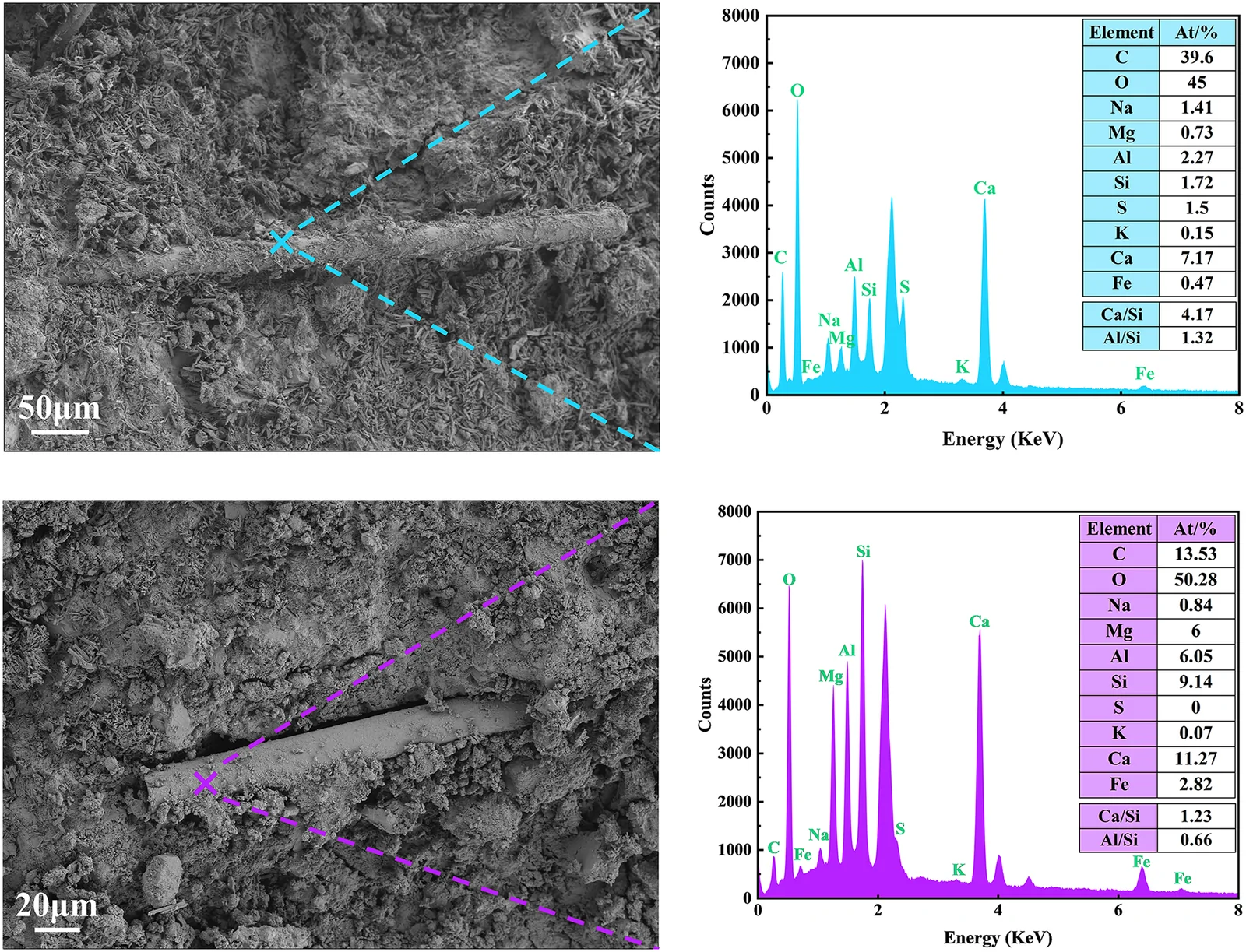
EDS test elements on the surfaces of PVAF and BF. (a) PVAF-EDS. (b) BF-EDS.

The elemental compositions on the surfaces of PVAF and BF, obtained by EDS point scanning, are shown in Fig 13. The main elements detected on the PVAF surface were C, O, and Ca, accompanied by a certain amount of S, indicating the presence of sulfate-related deposits such as gypsum and AFt at the interface. These deposits initially filled the pores between the fiber and matrix, strengthening the mechanical interlock. However, as expansive crystals continued to accumulate, they tended to induce local microcracks at the interface, compromising long-term stability. In contrast, the main elements on the BF surface were C, O, Ca, Si, and Al, with almost no S detected, indicating that the interfacial products primarily consisted of calcium salts and calcium aluminosilicates with a more stable structure. Further comparison showed that the Ca/Si (4.17) and Al/Si (1.32) ratios on the PVAF surface were significantly higher than those on the BF surface (1.23 and 0.66, respectively), suggesting that the PVAF interface was calcium rich but deficient in silicon and aluminum, leading to a relatively loose product structure. Conversely, the balanced distribution of Ca, Si, and Al at the BF interface facilitated the formation of a stable and dense calcium aluminosilicate gel [58]. This difference can be attributed to the hydroxyl groups on PVAF molecular chains, which exhibit strong adsorption capacity [59,60], allowing them to capture abundant sulfate ions, promote salt deposition, and initially densify the interface. However, this process also introduced a latent risk of expansion induced cracking. BF, as an inorganic aluminosilicate fiber, participated in the pozzolanic and gel forming reactions of the surrounding matrix under alkaline conditions [61], forming a stable and compact deposition layer. This layer not only enhanced the interfacial bond between the fiber and matrix but also effectively impeded further SO_4_^2-^ ingress. Such differences explain why FRMC incorporating BF exhibited superior long-term stability and resistance to deterioration under sulfate attack.

### 4.3 Phase analysis based on XRD

**Fig 14:**
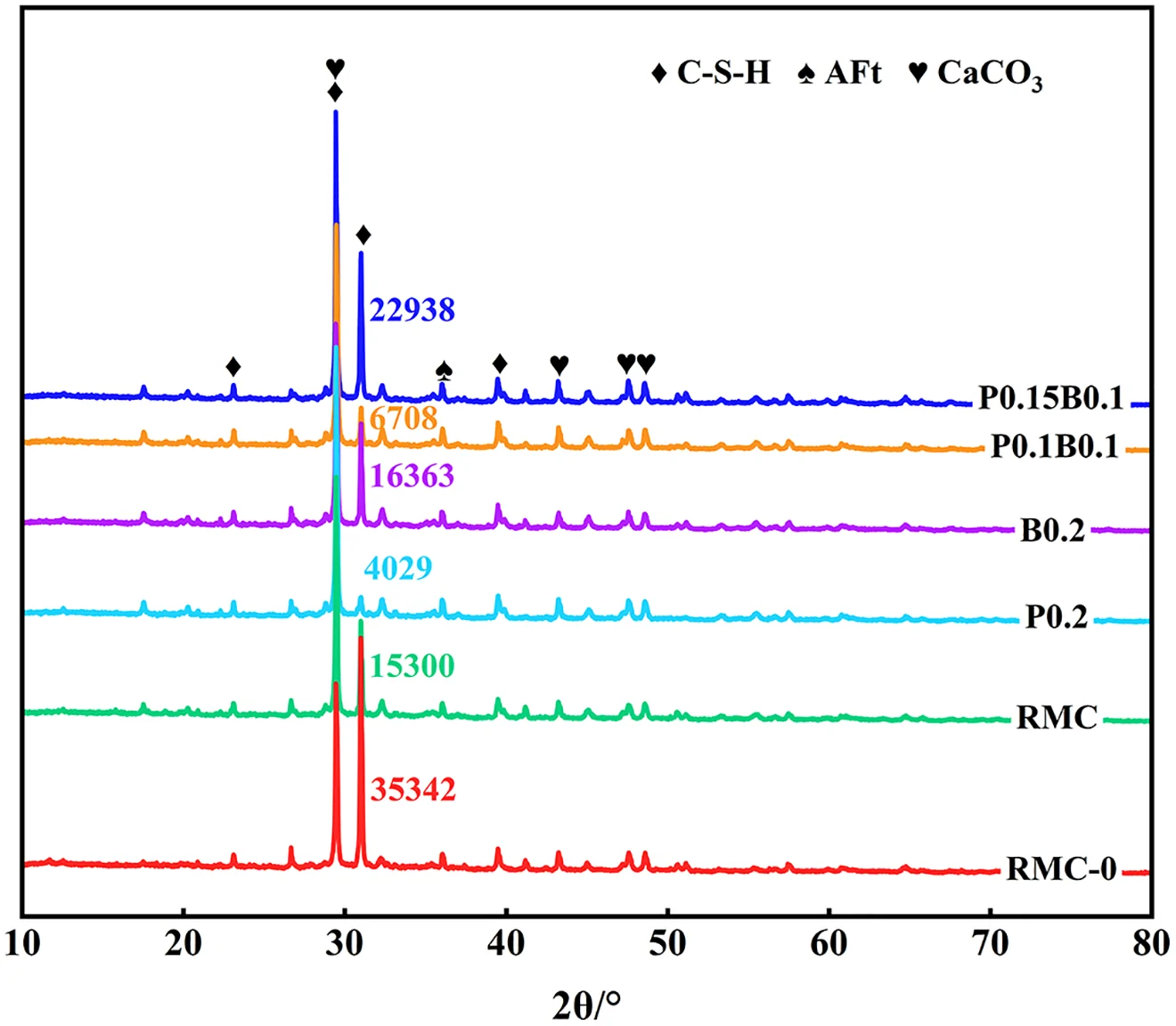
XRD patterns of specimens.

The XRD patterns of unexposed RMC (RMC-0) and those of RMC and FRMC after 60 cycles of sulfate drying–wetting are shown in Fig 14. The diffraction peaks of RMC-0 primarily corresponded to calcium silicate hydrate (C–S–H), ettringite (AFt), and calcite (CaCO_3_). After 60 cycles, no new diffraction peaks appeared in any specimen, indicating that no new crystalline phases were formed. However, the relative intensities of existing phases changed to varying degrees. It should be noted that C-S-H gel is a poorly crystalline or nearly amorphous phase, which generally appears in XRD patterns as a diffuse broad hump within the range of approximately 2θ = 26°–32°. In addition, this region may partially overlap with the characteristic peaks of CaCO_3_. Therefore, in this study, this diffuse hump region was taken as the analysis range for C-S-H, and the diffraction intensity at a representative position of approximately 2θ = 31° was selected and marked for semi-quantitative comparison among different samples. The results show that, after sulfate wet–dry cycling, the intensity of the diffuse hump near 2θ ≈ 31° decreased to varying degrees in all groups, indicating that the structure of the hydration products was partially damaged. This is mainly associated with Ca^2+^ leaching and C-S-H decalcification induced by SO_4_^2-^ ingress, which weakens the stability of the gel structure [62]. With respect to fiber type, the intensity attenuation in this region for the B0.2 group was smaller than that for the P0.2 group. This behavior can be attributed to the role of BF in crack control, as well as its possible surface reaction, nucleation, and interfacial regulation effects. Specifically, the presence of trace reactive SiO_2_ on the BF surface may participate in interfacial reactions with the surrounding matrix under alkaline conditions and provide nucleation sites for hydration products [63]. In contrast, PVAF mainly relied on its crack bridging capability to hinder ion penetration. As the cycles progressed, the surface of PVAF gradually aged, weakening the barrier effect and resulting in more pronounced degradation of the gel structure. The low hybrid fiber (P0.1B0.1) showed a similar reduction in the diffuse peak intensity to that of single PVAF reinforcement, corresponding to a significant decline in compressive strength at the macro scale. However, the moderate hybrid fiber (P0.15B0.1) effectively mitigated this attenuation, indicating that the PVAF–BF hybrid system exhibited a more favorable combined effect in crack control and microstructure stabilization. This improvement may be attributed to the combined influence of fiber interaction and the increase in total fiber content, which contributed to delaying the degradation of the gel structure. It should be emphasized that, because the above diffraction region may contain overlapping contributions from multiple phases, such as C-S-H and CaCO_3_, the related analysis mainly reflects relative variation trends. Combined with the SEM and MIP results, it can be further verified that the differences among different fiber systems in crack control and pore structure evolution are important factors responsible for the variation in the stability of hydration products. The AFt diffraction peak exhibited relatively minor fluctuation, reflecting a “dissolution–reprecipitation” dynamic equilibrium during sulfate attack. On one hand, in environments with high SO_4_^2-^ concentrations, AFt could dissolve or decompose due to insufficient Ca^2+^ or spatial confinement. On the other hand, Ca^2+^, Al^3+^, and SO_4_^2-^ in the solution could recombine to form new AFt crystals, which precipitated within pores or cracks. Initially formed AFt could fill capillary pores, temporarily enhancing compactness. However, as these products accumulated, their expansive properties generated interfacial stresses and microcracks, providing pathways for further SO_4_^2-^ ingress. The CaCO_3_ diffraction peak generally remained constant or slightly increased after sulfate drying–wetting cycles, primarily due to carbonation and the redeposition of carbonates following C–S–H decalcification. This process temporarily filled pores and improved matrix compactness but also consumed system alkalinity and calcium sources, reducing the buffering capacity against SO_4_^2-^ and, together with the expansive effect of AFt, accelerated the later stage deterioration.

The integrated analysis of the MIP, SEM-EDS, and XRD results indicates that the reinforcing effect of hybrid fibers in FRMC improves the durability of the material through multiscale coupled mechanisms. At the structural scale, the synergistic action of PVAF and BF effectively suppresses the initiation and propagation of microcracks, thereby reducing the penetration pathways for aggressive media. At the pore-structure scale, the controlled crack development further slows pore structure deterioration, facilitating pore refinement and the transformation of multiple harmful pores into harmless pores. At the product scale, the optimized pore-structure environment helps reduce the damage of sulfate attack products to the hydration gel, thereby improving the stability of hydration products. These multiscale effects are mutually coupled, forming a coupled enhancement mechanism of “crack resistance–pore structure optimization–hydration product protection”, which jointly improves the sulfate attack resistance of FRMC.

## 5 Conclusions

This study systematically investigated the enhancement mechanisms of PVAF and BF under sulfate drying-wetting cycles. The improvement of fibers on the sulfate resistance of RMC was evaluated by the mass change fraction, compressive strength and sulfate resistance coefficient. The effect micro-mechanisms of single and hybrid PVAF and BF on the sulfate resistance were elucidated by combining pore structure, microstructure, and phase composition results. The main conclusions are as follows:

(1) Under sulfate drying-wetting cycles, the mass of RMC initially increased and then decreased, falling below the initial value after 45 cycles due to mortar spalling, with a reduction of 1.87%. In contrast, the mass of FRMC specimens exhibited a continuous upward trend, as fibers restrained crack propagation and provided additional space for the accumulation of corrosion products such as gypsum and AFt, thereby contributing to the preservation of the overall structural integrity.
(2) Among all groups, the hybrid fiber system P0.15B0.1 (0.15% PVAF and 0.1% BF) exhibited the best sulfate resistance. Its compressive strength continuously increased and maintained a sulfate resistance coefficient of 0.854 after 60 cycles, whereas most other groups showed values lower than **0.75**.
(3) Fiber incorporation effectively delayed crack initiation and propagation. RMC without fibers exhibited the earliest cracking and severe surface deterioration, while BF showed better crack suppression than PVAF. The hybrid fiber further enhanced the synergistic crack resistance, with P0.15B0.1 showing the most effective retention of structural integrity.
(4) Sulfate drying–wetting cycles increased concrete porosity. Single fiber had limited effect on pore refinement, whereas P0.15B0.1 effectively optimized the pore size distribution. This fiber combination inhibited crack-induced pore coarsening and mitigated the degradation of hydration gel, thereby effectively improving the sulfate resistance of FRMC.

## 6 Discussion

The results of this study demonstrate that the hybrid incorporation of PVAF and BF can significantly enhance the sulfate attack resistance of red mud concrete through multiscale synergistic effects. Compared with previous studies that mainly focused on ordinary concrete or low-volume red mud systems, this study investigated red mud concrete with a high red mud content of 40% under sulfate wet–dry cycling, which represents a more severe condition and is closer to actual service environments. Therefore, this work extends the application research of red mud concrete in complex corrosive environments. Compared with single-fiber systems, the optimized hybrid fiber mixture (P0.15B0.1) achieved a better balance among crack control, pore structure optimization, and hydration product stability, which is a key factor in improving material durability. From an academic perspective, this study combines macroscopic performance evaluation with microstructural and phase analyses, including MIP, SEM-EDS, and XRD, and systematically reveals the coupled mechanism among crack resistance, pore structure evolution, and hydration gel protection. These findings provide new theoretical insights into the durability improvement of fiber-reinforced red mud concrete under sulfate attack. From an engineering application perspective, the results indicate that a rational hybrid fiber design can effectively improve the long-term durability of concrete in sulfate attack environments, providing important reference value for infrastructure in saline soil areas, coastal engineering, and industrial corrosive environments. The proposed optimal fiber proportion provides a basis for engineering material design and performance optimization.

However, this study still has certain limitations. First, this study mainly focused on a 40% RM replacement ratio and analyzed the improvement effect of fibers on the performance of RMC under this replacement level. Insufficient attention was paid to the interaction mechanism between RM and fibers under different RM replacement ratios. In particular, the influence of variations in RM content on the structure and action mechanism of the fiber–matrix interfacial transition zone has not been systematically investigated and requires further exploration over a wider range of replacement levels. Second, this study compared the performance differences between single-fiber and hybrid-fiber systems at a total fiber volume content of 0.2%. However, the optimal fiber dosage may differ among different systems, and systematic comparisons under their respective optimal dosages are still lacking. Therefore, the related conclusions need to be further verified and refined. In addition, the analysis of sulfate ion transport behavior in this study was mainly inferred based on pore structure characteristics and microstructural evolution, while direct supporting data from transport performance tests, such as RCPT or permeability tests, are still lacking.

Based on the above limitations, future research can be conducted in the following aspects. First, the ranges of RM replacement ratio and fiber dosage should be expanded to systematically reveal the fiber–matrix interactions under different parameter conditions and their influence on durability. Second, long-term service performance should be investigated under multifactor coupled conditions, such as the combined effects of sulfate attack with freeze–thaw cycles or carbonation. Third, RCPT or permeability tests should be introduced to provide a more systematic and quantitative evaluation of the ion migration characteristics of the material. Fourth, advanced characterization techniques and numerical simulation methods should be combined to further analyze the intrinsic relationship between the fiber reinforcement mechanism and microstructural evolution, thereby providing a more reliable theoretical basis for the design of highly durable cement-based materials.

## Supporting information

S1 Data Raw data. (XLSX)
